# Cross-sectional association of arsenic exposure with thyroid function in Bangladeshi children aged 5 to 7 years

**DOI:** 10.1186/s12940-025-01261-9

**Published:** 2026-01-07

**Authors:** Yingyue Ni, Prathiba Balakumar, Tariqul Islam, Syed Emdadul Haque, Mohammad H. Shahriar, Golam Sarwar, Alauddin Ahmed, Chunyu Liu, Brandon L. Pierce, Robert M. Sargis, Brian Jackson, Farzana Jasmine, Muhammad G. Kibriya, Habibul Ahsan, Maria Argos

**Affiliations:** 1 Department of Environmental Health, Boston University School of Public Health, Boston, MA United States; 2 Division of Epidemiology and Biostatistics, University of Illinois Chicago School of Public Health, Chicago, IL United States; 3 UChicago Research Bangladesh, Dhaka, Bangladesh; 4 Institute for Population and Precision Health, University of Chicago, Chicago, IL United States; 5 Department of Biostatistics, Boston University School of Public Health, Boston, MA United States; 6 Department of Public Health Sciences, University of Chicago, Chicago, IL United States; 7 Department of Medicine, College of Medicine, University of Illinois Chicago, Chicago, IL United States; 8 Department of Earth Sciences, Dartmouth College, Hanover, NH United States

**Keywords:** Arsenic, Thyroid function, Children’s health, Bangladesh, Environmental epidemiology

## Abstract

**Background:**

Arsenic is a pervasive environmental contaminant and a recognized global public health concern. Experimental evidence suggests that arsenic may disrupt endocrine signaling during critical developmental windows, yet epidemiologic data on its effects on thyroid function in early childhood remain limited.

**Methods:**

We investigated the cross-sectional association between arsenic exposure and free thyroxine (fT4) levels among 496 children aged 5 to 7 years enrolled in the Bangladesh Environmental Research in Children’s Health (BiRCH) cohort. Arsenic exposure was assessed using urinary total arsenic and toenail arsenic concentrations. Serum fT4 levels were measured by an enzyme-linked immunosorbent assay (ELISA) kit. Associations with fT4 were estimated using multivariable linear regression models adjusted for child age, sex, body mass index, and environmental tobacco smoke exposure.

**Results:**

The median urinary and toenail arsenic concentrations were 88.0 µg/L (interquartile range [IQR]: 127.4) and 1.7 µg/g (IQR: 2.0), respectively. Children in the highest quartile (Q4) of arsenic exposure had significantly higher fT4 levels compared to those in the lowest quartile (Q1), for both urinary (β = 0.09; 95% CI: 0.005–0.17) and toenail arsenic (β = 0.10; 95% CI: 0.03–0.17). A significant dose-response trend was observed across quartiles, suggesting a potential linear relationship.

**Conclusions:**

Our findings suggest that thyroid function may be a sensitive target of arsenic toxicity in early childhood. Longitudinal studies are necessary to assess the long-term effects of early-life arsenic exposure on thyroid function across the life course.

**Supplementary Information:**

The online version contains supplementary material available at 10.1186/s12940-025-01261-9.

## Background

Arsenic is a naturally occurring metalloid and a pervasive environmental contaminant with well-documented adverse health effects [1]. Human exposure occurs through multiple pathways, including contaminated drinking water, food, air, soil, and dust [2, 3]. Globally, an estimated 140 million people are chronically exposed to arsenic in drinking water at concentrations exceeding the World Health Organization (WHO) guideline of 10 µg/L [4].

Inorganic arsenic is classified as a Group 1 human carcinogen and has been linked to increased risks of skin, lung, and bladder cancers [1, 5]. Arsenic exposure has also been associated with a range of non-cancer outcomes, including neurodevelopmental, cardiovascular, reproductive, and metabolic disorders [6]. Emerging experimental evidence also suggests that arsenic acts as an endocrine disruptor, interfering with hormonal signaling pathways, including those of the thyroid axis [7, 8].

The thyroid gland plays a critical role in regulating metabolism, growth, and neurodevelopment through the production of thyroid hormones [8, 9]. These hormones are regulated by the hypothalamic-pituitary-thyroid (HPT) axis, which controls the synthesis and release of triiodothyronine (T3) and thyroxine (T4) [10]. T4, the primary hormone secreted by the thyroid, accounts for approximately 80% of total thyroid hormone production and serves as a precursor to the more biologically active T3 [11]. Free T4 (fT4) is a key indicator for assessing thyroid function in healthy populations [12], and shifts in fT4 levels may serve as a sensitive biomarker of population-level thyroid health.

Few epidemiologic studies have examined the relationship between arsenic exposure and thyroid hormone levels in childhood. An analysis of 4,855 participants aged 12 years and older from the National Health and Nutrition Examination Survey (NHANES) found that higher arsenic exposure was associated with lower levels of T4 and other thyroid hormones [13]. In Bangladesh, a pilot study among adolescents found a non-significant inverse association between groundwater arsenic concentration and free T4 levels [14].

Thyroid activity is markedly elevated during childhood [15], making this developmental stage particularly susceptible to arsenic-related disruption of thyroid function. However, data on arsenic-related thyroid disruption during early childhood remain limited. To address this gap, we evaluated the cross-sectional association between arsenic exposure (employing both urinary and toenail arsenic measurements to capture short- and long-term exposure) and fT4 levels in 496 Bangladeshi children aged 5 to 7 years participating in the Bangladesh Environmental Research in Children’s Health (BiRCH) cohort.

## Methods

### Study population

This study draws on data from the BiRCH cohort, which enrolled 500 mother-child pairs between 2014 and 2016 [16]. Children were aged 5 to 7 years at enrollment and were born to women participating in the Health Effects of Arsenic Longitudinal Study (HEALS), a prospective cohort based in Araihazar, Bangladesh, designed to investigate the health impacts of chronic arsenic exposure from drinking water [17]. We selected only one eligible child who met the age criteria per mother and was from a singleton birth. At enrollment, participants completed structured questionnaires and underwent clinical and laboratory assessments. This analysis includes 496 children, excluding four participants without available toenail arsenic data.

### Arsenic exposure assessment

Spot urine samples were collected in 50 mL acid-washed tubes and stored at − 20 °C in the field laboratory before being transported on dry ice to the Trace Metals Laboratory at Columbia University. Total urinary arsenic concentrations were measured using inductively coupled plasma-mass spectrometry (ICP-MS), with a limit of detection (LOD) of 2 µg/L [18]. To account for urine dilution, arsenic concentrations were adjusted for urinary creatinine [19], which was measured using a Jaffe reaction-based colorimetric method [20]. All samples had detectable levels of urinary arsenic and creatinine.

Toenail clippings were collected from each child by their caregiver, placed in prelabeled envelopes, and stored at room temperature. Samples were analyzed at the Dartmouth College Trace Element Analysis Core using ICP-MS [21]. Quality control procedures included calibration verification, duplicate and spike analyses, batch comparisons, and validation against standard reference materials. The LOD for toenail arsenic was 0.01 µg/g, and all samples were above the detection limit.

### Thyroxine assessment

Free thyroxine concentrations were measured in 50 µL serum samples collected from participating children at the time of enrollment. Samples were processed and stored at − 80 °C until analysis. Quantification of fT4 was performed using the CTK Free Thyroxin ELISA kit (E1026; CTK Biotech, Inc., Poway, CA), a competitive solid-phase enzyme-linked immunoassay enabling high-throughput and sensitive detection of hormone concentrations. Absorbance readings were obtained using the ADX-110 ELISA plate analyzer (CTK Biotech, Inc., Poway, CA), with automated plate washing performed using the ADX-120 ELISA plate washer (CTK Biotech, Inc., Poway, CA). Calibration curves were generated using CurveExpert 1.4, and all assay data were analyzed using Belysa Immunoassay Curve Fitting Software, Version 1 (Millipore, Germany), to ensure accurate and reproducible quantification. All assays were conducted at the Institute for Population and Precision Health Laboratory at the University of Chicago, following the manufacturer’s protocols. Quality control procedures included the use of internal standards and duplicate sample analysis.

### Covariates

Covariates were selected based on a priori knowledge. These included child sex, age, body mass index (BMI), father’s smoking status, and the presence of another current smoker in the household (excluding parents). All covariates except BMI were obtained from caregiver-completed questionnaires. BMI was calculated from height and weight measurements collected by trained study physicians. Maternal smoking was not included due to low prevalence (*n* = 1).

### Statistical analysis

Urinary and toenail arsenic concentrations were categorized into quartiles based on their distribution in the study sample, with the lowest quartile (Q1) serving as the reference group. Associations between arsenic exposure and fT4 levels were estimated using linear regression models, with arsenic quartiles included as indicator variables. Regression coefficients (β) represent the estimated differences in fT4 concentrations for each quartile compared with the reference group (Q1). To assess linear dose-response trends, we fit a model treating arsenic quartiles as an ordinal variable to obtain the p for trend. Three models were constructed: Model 1 adjusted for child sex and age; Model 2 additionally adjusted for BMI; and Model 3 further adjusted for paternal smoking and the presence of another smoker in the household. Models using urinary arsenic also included urinary creatinine as a covariate. There was no evidence of confounding by socioeconomic indicators; therefore, these variables were excluded to present the most parsimonious models. Effect modification by child sex and BMI was assessed by including a cross-product term between continuous arsenic exposure and the potential modifier. All analyses were conducted using R version 4.4.1 (R Foundation for Statistical Computing, Vienna, Austria).

## Results

Descriptive characteristics of the study sample are presented in Table 1. The median age of children at enrollment was 6.2 years (interquartile range [IQR]: 0.9), with a balanced sex distribution (50.6% male, 49.4% female). The mean BMI was 14.2 kg/m^2^ (kilograms per square meter) (standard deviation [SD]: 1.4). Among participants, 42.7% reported paternal smoking, and 18.1% reported the presence of another current smoker in the home.

**Table 1:** Baseline characteristics of BiRCH participants (*N* = 496)

Characteristic	*N* = 496
Child sex, *n* (%)	
Male	251 (50.6)
Female	245 (49.4)
Child age (years), median (IQR)	6.2 (0.9)
Child body mass index (kg/m^2^), mean ± SD	14.2 ± 1.4
Father’s current smoking status, *n* (%)	
Yes	212 (42.7)
No/Deceased	284 (57.3)
Other current smoker in the household, *n* (%)	
Yes	90 (18.1)
No	406 (81.9)
Urinary creatinine (mg/dL), median (IQR)	58.6 (45.1)
Urinary arsenic concentration (µg/L), median (IQR)	88.0 (127.4)
Toenail arsenic concentration (µg/g), median (IQR) ^*^	1.7 (2.0)
Serum fT4 (ng/dL), mean ± SD	1.25 ± 0.29

Median urinary arsenic concentration was 88.0 µg/L (IQR: 127.4), and median toenail arsenic concentration was 1.7 µg/g (IQR: 2.0). Urinary and toenail arsenic levels were moderately correlated (Spearman’s ρ = 0.57).

The distribution of fT4 levels is presented in Supplementary Fig. 1. The fT4 concentrations ranged from 0.65 to 2.21 ng/dL, with a mean of 1.25 (SD: 0.29) ng/dL. Reference ranges for fT4 vary considerably by assay and laboratory; therefore, clinical interpretation of these research-based results should be made with caution, as the assay was conducted in a research laboratory setting and was not intended for clinical diagnostic use. Approximately 18% of participants had fT4 levels below one available reference range for this age group (1.0–1.7 ng/dL), while 7% had elevated levels [22]. The distribution of fT4 levels across arsenic exposure quartiles is shown in Supplementary Fig. 2. Children in the highest exposure quartile (Q4) had higher median fT4 concentrations compared with those in the lowest quartile (Q1).

Associations between arsenic exposure and fT4 levels are summarized in Tables 2 and 3. In the fully adjusted model, children in the third quartile of urinary arsenic exposure had significantly higher fT4 levels compared to those in the lowest quartile (Q3 vs. Q1: β = 0.09, 95% CI: 0.02–0.17). Children in the fourth exposure quartile also had increased fT4 levels relative to the lowest quartile (Q4 vs. Q1: β = 0.09, 95% CI: 0.005–0.17). A significant linear dose-response trend was observed across quartiles (p for trend = 0.02).

Similar associations were observed for toenail arsenic concentrations. In the fully adjusted model, children in the highest exposure quartile had significantly elevated fT4 levels compared to those in the lowest quartile (Q4 vs. Q1: β = 0.10, 95% CI: 0.03–0.17), with a significant linear trend across quartiles (p for trend = 0.017).

**Table 2:** Association between urinary arsenic concentration (µg/L) and free thyroxine (fT4; ng/dL), *N* = 496

Arsenic, µg/L	Beta (95% CI)				*P* for trend
	Q1: 3.67–50.99	Q2: 51.05–87.95	Q3: 88.05–177.53	Q4: 180.93–1261.54	
Model 1^a^	Reference	0.05 (-0.02, 0.13)	0.10 (0.03, 0.18)	0.11 (0.03, 0.20)	0.004
Model 2^b^	Reference	0.05 (-0.02, 0.13)	0.10 (0.02, 0.18)	0.11 (0.03, 0.19)	0.005
Model 3^c^	Reference	0.05 (-0.03, 0.12)	0.09 (0.02, 0.17)	0.09 (0.005, 0.17)	0.02

Q1 to Q4 represent increasing quartiles of exposure, from the lowest to the highest. Each quartile has 124 children

^a^ Model 1: adjusted for urinary creatinine, child sex, and age

^b^ Model 2: adjusted for urinary creatinine, child sex, age, and BMI

^c^ Model 3: adjusted for urinary creatinine, child sex, age, BMI, and smoker in the household

**Table 3:** Association between toenail arsenic concentration (µg/g) and free thyroxine (fT4; ng/dL), *N* = 496

Arsenic, µg/g	Beta (95% CI)				*P* for trend
	Q1: 0.21–0.92	Q2: 0.93–1.69	Q3: 1.70–2.87	Q4: 2.88–12.63	
Model 1^a^	Reference	0.07 (-0.0005, 0.14)	0.05 (-0.02, 0.12)	0.12 (0.04, 0.19)	0.005
Model 2^b^	Reference	0.07 (-0.004, 0.14)	0.05 (-0.02, 0.12)	0.11 (0.04, 0.18)	0.007
Model 3^c^	Reference	0.07 (-0.002, 0.14)	0.04 (-0.03, 0.11)	0.10 (0.03, 0.17)	0.017

Q1 to Q4 represent increasing quartiles of exposure, from the lowest to the highest. Each quartile has 124 children

^a^ Model 1: adjusted for child sex and age

^b^ Model 2: adjusted for child sex, age, and BMI

^c^ Model 3: adjusted for child sex, age, BMI, and smoker in the household

Effect modification by child sex and BMI was assessed, but no significant interactions were detected (data not shown). Therefore, results were presented for the overall cohort.

## Discussion

To our knowledge, this is the first epidemiologic study to examine the association between arsenic exposure and thyroid hormone levels in young children. In a cohort of 496 Bangladeshi children aged 5 to 7 years, we observed that higher urinary and toenail arsenic concentrations were significantly associated with elevated fT4 levels. These associations exhibited a linear dose-response pattern, with children in the highest exposure quartile showing 0.09–0.10 ng/dL higher fT4 levels compared to those with the lowest exposure levels. Such population-level shifts in fT4 could move a substantial proportion of children into a range considered elevated for fT4. Elevated thyroid hormone levels in early life have been linked to altered growth trajectories, including reduced adult stature [23]. Later in life, modest increases in thyroid hormone levels may influence the risk of cardiovascular disease [24] and cancer [25]. Therefore, the observed shift in fT4 (0.09–0.10 ng/dL) may reflect biologically meaningful thyroid disruption during a critical developmental window, with potential long-term implications for health across the life course.

Our findings contribute to a growing but inconsistent body of literature on arsenic and thyroid function. Prior studies, including analyses of U.S. adults from NHANES, have reported inverse associations between arsenic exposure and T4 levels [26]. However, these discrepancies may reflect differences in exposure levels. In our study, the strongest magnitudes of association were observed at relatively high exposure levels (urinary arsenic ≥ 88.05 µg/L; toenail arsenic ≥ 2.88 µg/g), well above exposure levels observed in NHANES (urinary arsenic mean ± SD: 5.26 ± 0.09 µg/L) [27]. Additionally, associations may differ by developmental stage. A small pilot study of Bangladeshi adolescents (*n* = 32) with similarly high arsenic exposure did not detect associations with fT4 [14], possibly due to limited statistical power. Age-related differences in thyroid physiology may also play a role. The distinct etiologies and developmental implications of thyroid dysfunction across the life course underscore the importance of age-specific research [28, 29]. Our findings highlight the need for longitudinal studies that follow children into adolescence and adulthood to better understand the long-term consequences of early-life arsenic exposure.

The findings of this study provide some of the strongest evidence to date showing that arsenic exposure alters the HPT axis in children, an area of persistent scientific uncertainty. While limited in the extent of the analyses conducted, these findings underscore the need for a more comprehensive evaluation of the HPT axis to better understand the physiological consequences of arsenic exposure and the biological mechanisms underlying them. While a single observation of elevated fT4 does not allow for specific identification of the underlying impacts or modes of action, our results point to several biologically plausible pathways that warrant further investigation. In the context of low TSH, possible impacts include hyperactivation of thyroid hormone receptor signaling or reduced binding of thyroxine to thyroid-binding globulin or other transport proteins. Alternatively, in the presence of normal or elevated TSH, thyroid hormone resistance or impaired conversion of T4 to T3 may be implicated [30]. Previous experimental work by Davey et al. showed that arsenic disrupts thyroid hormone receptor (TR)-mediated gene regulation, particularly by altering the expression of TR-regulated genes such as type I deiodinase (DIO1) [7]. Our findings align with these observations and suggest that arsenic-induced disruption of TR signaling could lead to thyroid hormone resistance, resulting in compensatory elevation of free and total T4. Furthermore, arsenic-mediated impacts on DIO1 expression may impair the conversion of T4 to T3, resulting in elevated T4. While preliminary, the findings reported herein provide further critical support to the emerging evidence that arsenic is a likely disruptor of the HPT axis with potential impacts on critical aspects of child health and development.

This study has several strengths. The relatively large sample size (*n* = 496), use of both short-term (urinary) and long-term (toenail) biomarkers of arsenic exposure, and robust statistical modeling enhance the validity of our findings. The wide range of arsenic exposure in the study sample, spanning moderate to high levels, enabled us to evaluate a more comprehensive dose-response profile. The use of toenail arsenic as a biomarker of chronic exposure is particularly novel in the context of endocrine research in children, as it reflects exposure over the prior 5 to 18 months and may offer greater stability than urinary measures [31, 32], which capture short-term exposure over the prior 2 to 3 days [6]. Moreover, the consistency of findings across both exposure metrics strengthens the robustness of our results and suggests that each measure reasonably reflects chronic inorganic arsenic exposure from drinking water.

However, several limitations should be acknowledged. The cross-sectional design limits our ability to infer the temporal relationship between arsenic exposure and fT4, limiting causal inference [33]. Nevertheless, despite the cross-sectional design, we observed consistent associations using both short and longer-term arsenic exposure measures, which increases confidence in the temporality of the associations. Residual confounding from unmeasured factors such as iodine status, dietary intake, or co-exposure to other environmental toxicants cannot be ruled out. Iodine, an essential component of thyroid hormones, was not measured in this study and may influence fT4 levels. Over one-third of Bangladeshi children are reported to have iodine deficiency, which may alter thyroid hormone function and potentially modify responses to arsenic exposures [34]. Iodine deficiency is typically characterized by lower serum T4 [35], which may explain the high proportion of children observed at the lower end of the serum fT4 distribution in our study population. Future studies should evaluate potential effect modification by iodine status. Additionally, we did not assess other key thyroid-related hormones such as T3, thyroid-stimulating hormone (TSH), or thyrotropin-releasing hormone (TRH), which would provide a more comprehensive picture of thyroid function and regulatory feedback mechanisms.

Our findings contribute to the limited body of evidence on the impact of arsenic exposure on thyroid hormone regulation during early childhood, a sensitive developmental period where hormonal disruption may have lasting effects on growth, neurodevelopment, and metabolic function. These results carry important public health implications, including the potential for increased risk of neurodevelopmental delays, cognitive impairments, and metabolic disorders later in life. They underscore the urgent need for child-specific public health guidelines addressing arsenic exposure and highlight the importance of sustained efforts to mitigate exposure in affected communities, such as through subsidized well water testing and remediation initiatives.

## Conclusions

This study provides novel evidence that high levels of arsenic exposure are positively associated with higher fT4 concentrations in children aged 5 to 7 years living in a region with elevated groundwater arsenic concentrations. These findings suggest that thyroid function may be a sensitive target of arsenic toxicity during early childhood, a critical period for growth and development. Future research should incorporate additional thyroid-related biomarkers, assess co-exposures to other metals and relevant environmental exposures, and evaluate iodine status to better understand the mechanisms underlying arsenic-induced thyroid dysfunction. Longitudinal studies are needed to determine the persistence and clinical relevance of these hormonal changes across the life course.

## Supplementary Information

Supplementary Material 1.

## Data Availability

The data supporting the findings of this study are available from the corresponding author upon reasonable request.

## Abbreviations

fT4: Free thyroxine
T4: Thyroxine
T3: Triiodothyronine
TSH: Thyroid-stimulating hormone
TRH: Thyrotropin-releasing hormone
HPT: Hypothalamic-pituitary-thryoid
DIO1: Type I deiodinase
BiRCH: Bangladesh Environmental Research in Children’s Health
HEALS: Health Effects of Arsenic Longitudinal Study
NHANES: National Health and Nutrition Examination Survey
WHO: World Health Organization
ELISA: Enzyme-linked immunosorbent assay
ICP-MS: Inductively coupled plasma-mass spectrometry
LOD: Limit of detection
IQR: Interquartile range
SD: Standard deviation
BMI: Body mass index
