# Supplementary material for: Cross-sectional association of arsenic exposure with thyroid function in Bangladeshi children aged 5 to 7 years
Source: Environ Health. 2026 Jan 7;25:9. doi: 10.1186/s12940-025-01261-9 (PMC12870816; doi:10.1186/s12940-025-01261-9)
Supplement: Supplementary file 1 — Supplementary Material 1. [file 12940_2025_1261_MOESM1_ESM.docx]

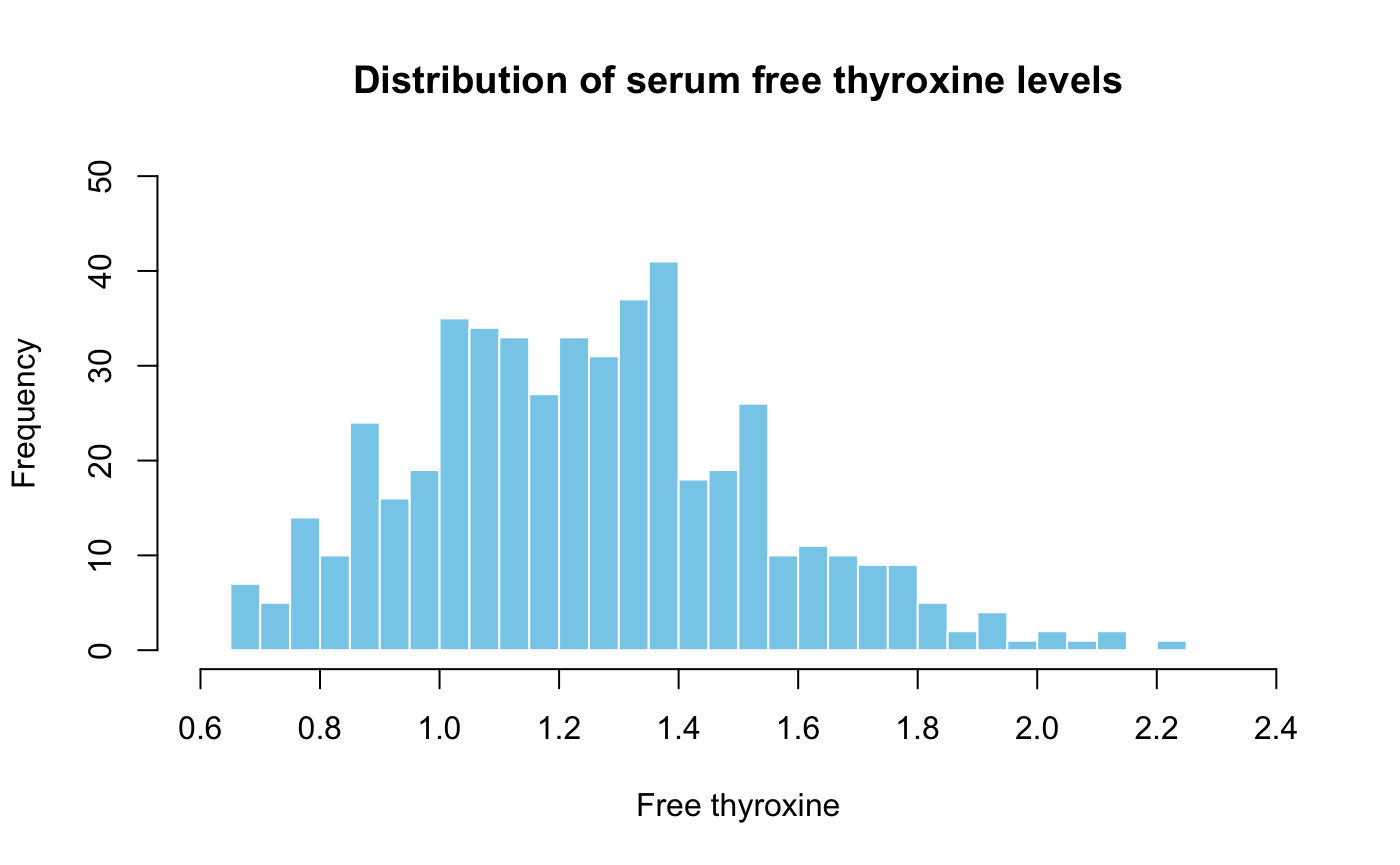


**Supplementary Figure 1. Distribution of free thyroxine (fT4; ng/dL) levels, N=496**

The distribution of the serum fT4 levels is shown.


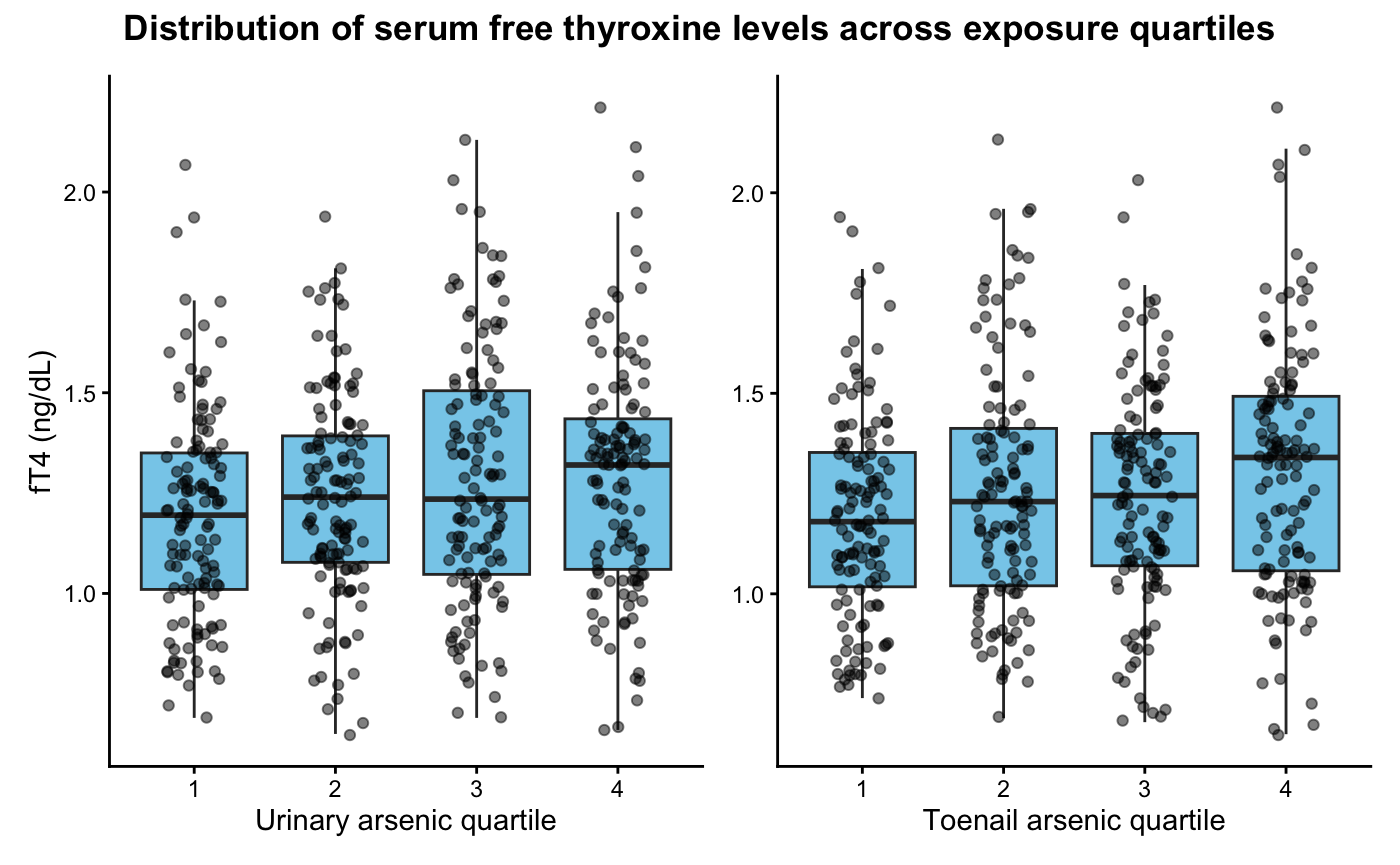


**Supplementary Figure 2. Distribution of free thyroxine (fT4; ng/dL) levels across arsenic exposure quartiles, N=496**

The distribution of the serum fT4 levels across urinary and toenail arsenic quartiles is shown.


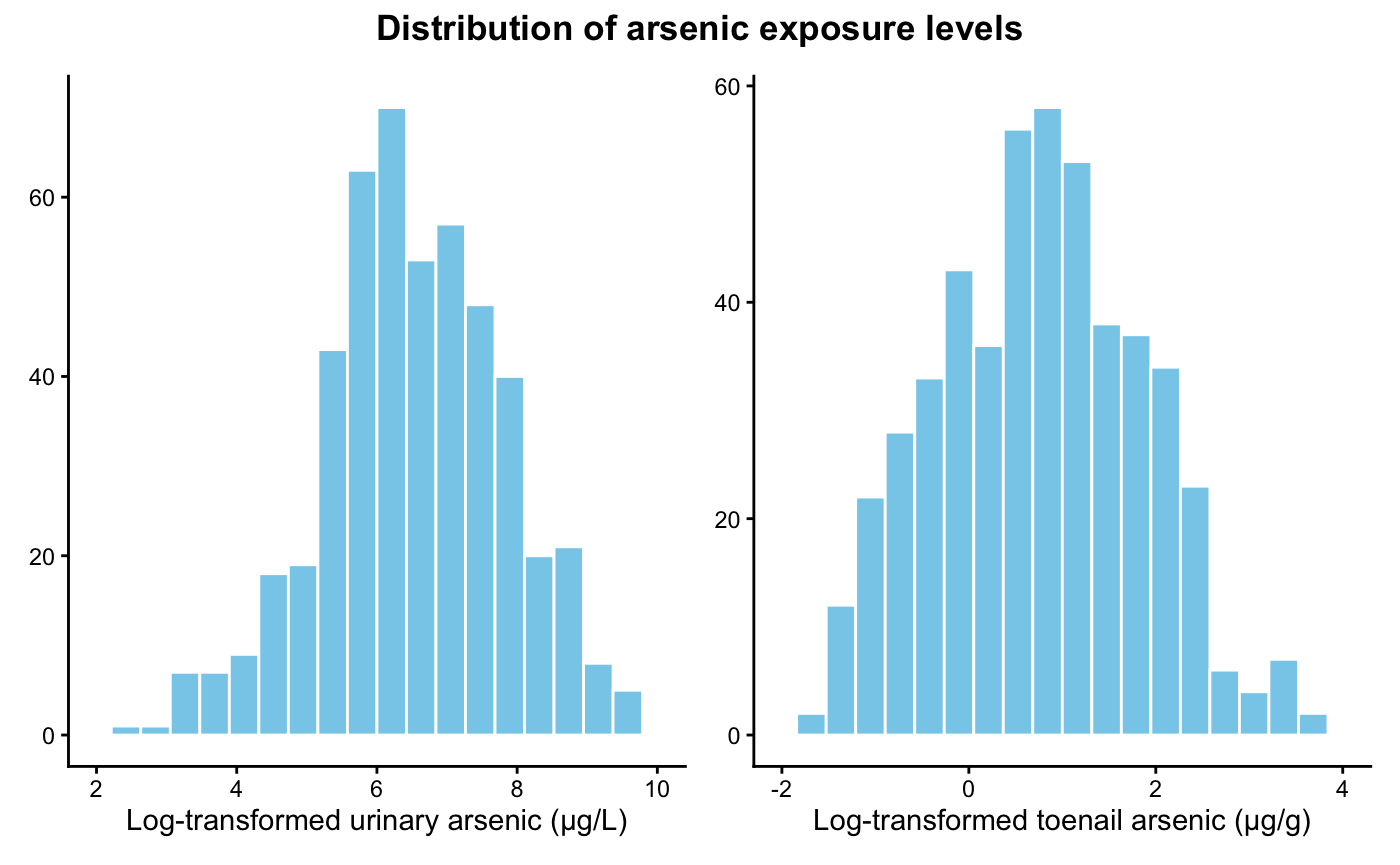


**Supplementary Figure 3. Distribution of arsenic exposure levels, N=496**

The distribution of urinary total arsenic and toenail arsenic concentrations is shown.
